# Supplementary material for: Association between Five Common Plasminogen Activator Inhibitor-1 (PAI-1) Gene Polymorphisms and Colorectal Cancer Susceptibility
Source: Int J Mol Sci. 2020 Jun 18;21(12):4334. doi: 10.3390/ijms21124334 (PMC7352892; doi:10.3390/ijms21124334)
Supplement: Supplementary file 1 [file ijms-21-04334-s001.pdf]

Supplementary Table 1. ***PAI-1*** **gene polymorphism frequencies and AORs in colon and rectal cancer cases and controls**

Characteristic	Controls (n=416)	Colon (n=268)	AOR (95% CI)	P^a	P^b	Rectum (n=191)	AOR (95% CI)	P^a	P^b
PAI-1 -844G>A									
GG	136 (32.7)	91 (34.0)	1.000 (reference)			63 (33.0)	1.000 (reference)		
GA	199 (47.8)	131 (48.9)	0.905 (0.628 - 1.304)	0.59	0.99	99 (51.8)	1.052 (0.697 - 1.589)	0.81	0.81
AA	81 (19.5)	46 (17.2)	0.800 (0.493 - 1.299)	0.37	0.61	29 (15.2)	0.794 (0.442 - 1.429)	0.44	0.68
Dominant (GG vs GA + AA)			0.872 (0.619 - 1.231)	0.44	0.59		0.978 (0.659 - 1.451)	0.91	0.91
Recessive (GG + GA vs AA)			0.894 (0.587 - 1.362)	0.60	0.94		0.782 (0.478 - 1.280)	0.33	0.56
PAI-1-675 4G>5G									
4G4G	180 (43.3)	109 (40.7)	1.000 (reference)			62 (32.5)	1.000 (reference)		
4G5G	180 (43.3)	108 (40.3)	1.018 (0.711 - 1.458)	0.92	0.99	98 (51.3)	1.522 (1.012 - 2.288)	0.04	0.22
5G5G	56 (13.5)	51 (19.0)	1.525 (0.942 - 2.468)	0.09	0.22	31 (16.2)	1.563 (0.874 - 2.798)	0.13	0.61
Dominant (4G4G vs 4G5G + 5G5G)			1.130 (0.811 - 1.575)	0.47	0.59		1.519 (1.033 - 2.234)	0.03	0.17
Recessive (4G4G + 4G5G vs 5G5G)			1.492 (0.965 - 2.307)	0.07	0.18		1.207 (0.718 - 2.030)	0.48	1.00
PAI-1 +43G>A									
GG	335 (80.5)	227 (84.7)	1.000 (reference)			148 (77.5)	1.000 (reference)		
GA	75 (18.0)	38 (14.2)	0.768 (0.491 - 1.203)	0.25	0.99	32 (16.8)	1.155 (0.717 - 1.861)	0.55	0.81
AA	6 (1.4)	3 (1.1)	0.717 (0.138 - 3.711)	0.69	0.86	11 (5.8)	0.511 (0.059 - 4.410)	0.54	0.68
Dominant (GG vs GA + AA)			0.766 (0.495 - 1.100)	0.23	0.58		1.113 (0.697 - 1.777)	0.66	0.91
Recessive (GG + GA vs AA)			0.769 (0.149 - 3.966)	0.75	0.94		0.500 (0.058 - 4.298)	0.53	0.66
PAI-1 +9785G>A									
GG	383 (92.1)	245 (91.4)	1.000 (reference)			172 (90.1)	1.000 (reference)		
GA	31 (7.5)	23 (8.6)	1.006 (0.538 - 1.879)	0.99	0.99	19 (9.9)	1.135 (0.567 - 2.268)	0.72	0.81
AA	2 (0.5)	0 (0.0)	N/A	1.00	1.00	0 (0.0)	N/A	1.00	1.00
Dominant (GG vs GA + AA)			0.934 (0.504 - 1.732)	0.83	0.83		1.053 (0.531 - 2.088)	0.88	0.91
Recessive (GG + GA vs AA)			N/A	1.00	1.00		N/A	1.00	1.00
PAI-1 +11053T>G									
TT	107	82 (30.6)	1.000 (reference)			51 (26.7)	1.000 (reference)		

	(25.7)								
TG	204 (49.0)	135 (50.4)	0.906 (0.619 - 1.328)	0.61	0.99	106 (55.5)	1.171 (0.750 - 1.827)	**0.49**	**0.81**
GG	105 (25.2)	51 (19.0)	0.594 (0.370 - 0.954)	**0.03**	0.16	34 (17.8)	0.721 (0.417 - 1.247)	**0.24**	**1.00**
Dominant (TT vs TG+GG)			0.798 (0.557 - 1.144)	0.22	0.58		1.024 (0.671 - 1.563)	**0.91**	**0.91**
Recessive (TT+TG vs GG)			0.667 (0.446 - 0.990)	**0.05**	0.18		0.690 (0.437 - 1.089)	**0.11**	**0.56**

***The adjusted odds ratio based on risk factors, such as age, gender, hypertension, and diabetes mellitus.**

[a] *P*-value calculated by multiple logistic regression analysis.

[b] False-positive discovery rate (FDR)-adjusted *P*-value.

Supplementary Table 2. Differences in various clinical parameters according to *PAI-1* gene polymorphisms in colorectal cancer patients

	BMI (kg/m^2)		Chol (mg/dL)		Folate (ng/dL)		Hcy (μmol/L)		HDL (mg/dL)		TG (mg/dL)		VB_{12} (mg)	
**Genotype**	N	mean ±SD	N	mean ±SD	N	mean ±SD	N	mean ±SD	N	mean ±SD	N	mean ±SD	N	mean ±SD
PAI-1- 844G>A														
GG	233	23.36 ±3.17	193	184.70 ±37.44	256	8.00 ±5.31	258	9.45 ±3.48	154	41.25 ±11.49	258	144.61 ±89.52	140	816.99 ±1005.02
GA	356	23.50 ±3.36	284	186.39 ±37.36	384	8.76 ±8.58	385	10.47 ±7.21	240	44.71 ±14.59	393	129.64 ±81.10	208	808.77 ±804.69
AA	121	23.88 ±3.29	109	189.81 ±44.84	140	8.66 ±6.65	140	10.32 ±7.10	84	45.10 ±11.00	142	133.59 ±75.05	85	642.27 ±277.00
P		0.37		0.55		0.42		0.504[a]		0.02		0.08		0.22
PAI-1 -675														
4G4G	277	23.67 ±3.34	244	185.70 ±40.85	303	8.61 ±6.05	305	10.07 ±5.83	174	43.61 ±14.25	314	131.69 ±85.23	187	726.60 ±572.65
4G5G	321	23.40 ±3.16	261	187.54 ±37.74	350	8.69 ±8.82	350	10.30 ±7.20	220	44.59 ±13.00	352	136.66 ±83.38	189	792.49 ±791.02
5G5G	112	23.50 ±3.54	81	185.36 ±36.55	127	7.69 ±5.16	128	9.67 ±3.74	84	41.37 ±10.78	127	139.91 ±76.95	57	904.25 ±1354.80
P		0.60		0.84		0.40		0.61		0.16		0.58		0.37[a]
PAI-1 43G>A														
GG	23	23.51 ±3.29	476	186.39 ±39.61	645	8.49 ±7.47	647	10.22 ±6.57	400	43.50 ±13.53	654	136.79 ±86.34	353	777.13 ±769.09
GA	111	23.60 ±3.34	103	184.47 ±33.09	128	8.56 ±6.64	128	9.65 ±4.23	76	44.35 ±11.05	131	127.34 ±66.86	75	797.37 ±1003.00
AA	5	23.38 ±2.44	7	221.29 ±53.46	7	7.62 ±4.21	8	8.76 ±2.23	51	51.05 ±5.30	8	135.75 ±34.03	5	613.60 ±100.22
P		0.96		0.05		0.95		0.53		0.64		0.50		0.88
PAI-1 9785G>A														
GG	655	23.53 ±3.31	532	186.45 ±38.49	721	8.44 ±7.43	724	10.23 ±6.40	447	43.89 ±13.10	736	134.08 ±82.35	399	784.83 ±838.18
GA	53	23.49 ±3.14	52	185.17 ±42.72	57	9.25 ±5.84	57	8.65 ±2.94	30	40.37 ±13.76	55	145.07 ±82.35	32	692.56 ±297.06
AA	2	22.35 ±0.50	2	226.00 ±18.38	2	6.64 ±0.91	2	9.26 ±0.16	1	40.40 ±0.00	2	281.50 ±259.51	2	943.50 ±447.60
P		0.88		0.35		0.68		0.18		0.36		0.31[a]		0.79
PAI-1 11053T>G														
TT	193	23.45 ±3.28	161	186.85 ±39.43	200	8.85 ±9.51	202	9.78 ±3.64	129	42.85 ±11.45	218	130.50 ±77.36	111	940.17 ±1215.16
TG	366	23.60 ±3.39	289	186.39 ±37.66	402	8.40 ±6.50	403	10.09 ±5.95	241	44.48 ±14.95	402	137.05 ±87.75	214	697.69 ±431.39
GG	151	23.41 ±3.05	136	186.19 ±40.91	178	8.31 ±6.16	178	10.53 ±8.67	108	42.82 ±10.42	173	136.91 ±79.04	108	773.44 ±845.49
P		0.78		0.99		0.72		0.50		0.840[a]		0.62		0.17[a]

Note: BMI = body mass index, Chol = total cholesterol, Hcy = homocysteine, HDL = high-density lipoprotein, TG = triglycerides, VB_{12} = vitamin B_{12}

[a] Analysis was performed using the Kruskal-Wallis test of differences in the biochemical parameters between *PAI-1* gene polymorphisms in colorectal cancer patients and controls.

Supplementary Table 3. Multivariate survival analysis according to *PAI-1* gene polymorphisms in colorectal cancer patients

Genotype	Patients (n=459)	Overall survival				Relapse-free survival			
		Death (n=93)	Adjusted HR (95% CI)	P^a	P^b	Relapse (n=78)	Adjusted HR (95% CI)	P^a	P^b
PAI-1 -844									
GG	154 (33.6)	33 (35.5)	1.000 (reference)			30 (38.5)	1.000 (reference)		
GA	230 (50.1)	47 (50.5)	0.946 (0.551-1.622)	0.84	0.93	40 (51.3)	1.011 (0.573-1.782)	0.97	0.97
AA	75 (16.3)	13 (14.0)	0.842 (0.348-2.039)	0.70	0.76	8 (10.3)	0.646 (0.244-1.707)	0.38	0.76
Dominant (GG vs GA + AA)			0.957 (0.571-1.606)	0.87	0.87		0.988 (0.573-1.704)	0.97	0.97
Recessive (GG + GA vs AA)			0.946 (0.439-2.037)	0.89	0.89		0.812 (0.351-1.876)	0.63	0.83
PAI-1-675 4G5G									
4G4G	171 (37.3)	36 (38.7)	1.000 (reference)			22 (28.2)	1.000 (reference)		
4G5G	206 (44.9)	38 (40.9)	1.177 (0.677-2.045)	0.57	0.93	41 (52.6)	1.172 (0.645-2.128)	0.60	0.91
5G5G	82 (17.9)	19 (20.4)	0.896 (0.445-1.804)	0.76	0.76	15 (19.2)	0.949 (0.454-1.986)	0.89	0.89
Dominant (4G4G vs 4G5G + 5G5G)			1.053 (0.637-1.741)	0.84	0.87		1.189 (0.683-2.067)	0.54	0.76
Recessive (4G4G + 4G5G vs 5G5G)			0.865 (0.465-1.610)	0.65	0.89		1.058 (0.570-1.963)	0.86	0.86
PAI-1 +43									
GG	375 (81.7)	75 (80.6)	1.000 (reference)			60 (76.9)	1.000 (reference)		
GA	70 (15.3)	16 (17.2)	0.972 (0.526-1.794)	0.93	0.93	17 (21.8)	1.111 (0.613-2.015)	0.73	0.91
AA	14 (3.1)	2 (2.2)	8.551 (1.833-39.89)	**0.01**	0.02	1 (1.3)	12.71 (1.330-121.3)	**0.03**	0.11
Dominant (GG vs GA + AA)			1.108 (0.619-1.982)	0.73	0.87		1.164 (0.650-2.085)	0.61	0.76
Recessive (GG + GA vs AA)			9.330 (2.043-42.61)	**0.01**	**0.02**		11.97 (1.433-100.0)	**0.02**	0.09
PAI-1 +9785									
GG	417 (90.8)	88 (94.6)	1.000 (reference)			74 (94.9)	1.000 (reference)		
GA	42 (9.2)	5 (5.4)	0.337 (0.101-1.119)	0.08	0.38	4 (5.1)	0.393 (0.116-1.332)	0.13	0.36
AA	0 (0.0)	0 (0.0)	N/A			0 (0.0)	N/A		
Dominant (GG vs GA + AA)			0.337 (0.101-1.119)	0.08	0.38		0.393 (0.116-1.332)	0.13	0.67
Recessive (GG + GA vs AA)			N/A				N/A		
PAI-1 +11053									
TT	133 (29.0)	36 (38.7)	1.000 (reference)			28 (35.9)	1.000 (reference)		
TG	241 (52.5)	39 (41.9)	0.904 (0.521-1.571)	0.72	0.93	35 (44.9)	0.644 (0.359-1.158)	0.14	0.36
GG	85 (18.5)	18 (19.4)	1.159 (0.583-2.307)	0.67	0.76	15 (19.2)	1.118 (0.551-2.266)	0.76	0.89
Dominant (TT vs TG+GG)			0.904 (0.552-1.481)	0.69	0.87		0.760 (0.454-1.273)	0.30	0.74

Recessive (TT+TG vs GG)	1.070 (0.578-1.981)	0.83	0.89	1.247 (0.658-2.363)	0.50	0.83

*The adjusted hazard ratio based on risk factors, such as age, gender, hypertension, diabetes mellitus, tumor size, tumor differentiation, chemotherapy, and TNM stage.

[a] *P*-value calculated by the Cox proportional hazard regression analysis.

[b] False-positive discovery rate (FDR)-adjusted *P*-value.

Supplementary Table 4. Results of stepwise analysis with Cox propotional-hazard regression in colorectal cancer survival

Covariate	β	SE	HR (95% CI)	*P*
**Overall survival with *PAI-1* +43 GG vs. AA**				
Sex	-0.633	0.261	0.531 (0.318-0.886)	0.02
TNM stage	1.628	0.201	5.095 (3.434-7.558)	<0.01
PAI-1 +43 GG vs. AA	2.143	0.754	8.524 (1.946-37.338)	0.01
**Overall survival with *PAI-1* +43 GG+GA vs. AA**				
TNM stage	1.559	0.178	4.753 (3.357-6.731)	<0.01
PAI-1 +43 GG+GA vs. AA	2.203	0.754	9.051 (2.064-39.681)	0.01
**Relapse-free survival with *PAI-1* +43 GG vs. AA**				
Sex	-0.622	0.289	0.537 (0.305-0.946)	0.03
Lymph node metastasis	0.613	0.232	1.845 (1.171-2.909)	0.01
TNM stage	1.889	0.282	6.616 (3.804-11.505)	<0.01
PAI-1 +43 GG vs. AA	2.603	1.109	13.497 (1.535-118.716)	0.02
**Relapse-free survival with *PAI-1* +43 GG+GA vs. AA**				
Lymph node metastasis	0.432	0.204	1.541 (1.032-2.299)	0.03
TNM stage	1.685	0.231	5.392 (3.427-8.483)	<0.01
PAI-1 +43 GG+GA vs. AA	2.555	1.083	12.876 (1.541-107.603)	0.02

Note: β, regression coefficient; SE, standard error; HR, hazard ratio; CI, confidence interval.

Supplementary Table 5. Colorectal cancer incidence by interactions with environmental factors such as age, gender, hypertension, diabetes mellitus, smoking, folate, homocysteine, BMI, VB12, triglycerides, cholesterol, HDL and LDL.

Characteristic	*PAI-1* -844 GG	*PAI-1* -844 GA+AA	*PAI-1* -675 4G4G	*PAI-1* -675 4G5G+5G5G	*PAI-1* 43 GG	*PAI-1* 43 GA+AA	*PAI-1* 9785 GG	*PAI-1* 9785 GA+AA	*PAI-1* 11053 TT	*PAI-1* 11053 TG+GG
Age										
<61	1.000 (reference)	1.066 (0.692 - 1.641)	1.000 (reference)	1.942 (1.247 - 3.024)	1.000 (reference)	0.889 (0.528 - 1.495)	1.000 (reference)	0.598 (0.283 - 1.265)	1.000 (reference)	0.812 (0.509 - 1.295)
≥61	0.778 (0.453 - 1.335)	0.660 (0.420 - 1.037)	1.085 (0.666 - 1.767)	1.207 (0.767 - 1.897)	0.749 (0.536 - 1.046)	0.733 (0.431 - 1.246)	0.675 (0.492 - 0.925)	1.521 (0.675 - 3.431)	0.747 (0.418 - 1.334)	0.578 (0.353 - 0.945)
Gender										
Male	1.000 (reference)	0.906 (0.565 - 1.452)	1.000 (reference)	1.313 (0.824 - 2.093)	1.000 (reference)	1.029 (0.568 - 1.862)	1.000 (reference)	1.306 (0.483 - 3.532)	1.000 (reference)	0.793 (0.473 - 1.329)
Female	0.668 (0.372 - 1.200)	0.617 (0.370 - 1.031)	0.620 (0.358 - 1.071)	0.884 (0.544 - 1.437)	0.713 (0.498 - 1.022)	0.595 (0.351 - 1.007)	0.716 (0.510 - 1.004)	0.721 (0.364 - 1.427)	0.459 (0.237 - 0.889)	0.513 (0.294 - 0.894)
Hypertension										
No	1.000 (reference)	1.231 (0.790 - 1.918)	1.000 (reference)	1.694 (1.093 - 2.627)	1.000 (reference)	0.980 (0.575 - 1.669)	1.000 (reference)	1.395 (0.670 - 2.907)	1.000 (reference)	0.734 (0.458 - 1.177)
Yes	3.057 (1.767 - 5.289)	2.053 (1.297 - 3.249)	2.861 (1.747 - 4.685)	**3.352 (2.134 - 5.265)**	2.487 (1.768 - 3.498)	2.143 (1.240 - 3.705)	2.508 (1.824 - 3.448)	1.870 (0.804 - 4.350)	1.588 (0.887 - 2.844)	1.596 (0.972 - 2.620)
Diabetes mellitus										
No	1.000 (reference)	1.059 (0.755 - 1.486)	1.000 (reference)	1.464 (1.051 - 2.039)	1.000 (reference)	0.780 (0.516 - 1.179)	1.000 (reference)	1.139 (0.629 - 2.062)	1.000 (reference)	0.901 (0.629 - 1.291)
Yes	3.978 (1.968 - 8.041)	2.210 (1.371 - 3.563)	3.166 (1.760 - 5.694)	3.325 (2.044 - 5.409)	2.410 (1.636 - 3.550)	**3.599 (1.396 - 9.279)**	2.767 (1.906 - 4.017)	1.616 (0.474 - 5.506)	2.617 (1.288 - 5.316)	2.412 (1.473 - 3.950)
Smoking										
No	1.000 (reference)	0.927 (0.649 - 1.323)	1.000 (reference)	1.351 (0.962 - 1.898)	1.000 (reference)	0.798 (0.516 - 1.234)	1.000 (reference)	1.227 (0.634 - 2.377)	1.000 (reference)	0.766 (0.530 - 1.108)
Yes	0.454 (0.236 - 0.874)	0.431 (0.252 - 0.738)	0.428 (0.223 - 0.824)	0.595 (0.359 - 0.986)	0.452 (0.300 - 0.680)	0.517 (0.255 - 1.049)	0.505 (0.343 - 0.742)	0.372 (0.133 - 1.036)	0.238 (0.108 - 0.524)	0.364 (0.212 - 0.625)
*Folate										
>3.8 nmol/L	1.000 (reference)	1.017 (0.715 - 1.447)	1.000 (reference)	1.455 (1.033 - 2.048)	1.000 (reference)	0.864 (0.562 - 1.330)	1.000 (reference)	1.075 (0.581 - 1.989)	1.000 (reference)	1.125 (0.771 - 1.640)
≤3.8 nmol/L	6.178 (2.590 - 14.737)	2.672 (1.476 - 4.837)	2.578 (1.207 - 5.506)	**5.163 (2.786 - 9.570)**	3.541 (2.174 - 5.768)	2.712 (0.864 - 8.519)	3.311 (2.095 - 5.232)	N/A	5.822 (2.084 - 16.264)	3.394 (1.846 - 6.241)
**Homocysteine										
<13.2 μmol/L	1.000 (reference)	0.870 (0.617 - 1.225)	1.000 (reference)	1.621 (1.156 - 2.272)	1.000 (reference)	0.726 (0.468 - 1.128)	1.000 (reference)	1.071 (0.580 - 1.976)	1.000 (reference)	1.132 (0.779 - 1.644)
≥13.2 μmol/L	1.138 (0.486 - 2.662)	1.403 (0.789 - 2.497)	1.997 (0.948 - 4.209)	1.856 (1.034 - 3.331)	1.453 (0.902 - 2.342)	1.857 (0.693 - 4.978)	1.605 (1.029 - 2.504)	0.956 (0.117 - 7.839)	2.388 (0.968 - 5.891)	1.631 (0.888 - 2.997)
BMI										
< 25 kg/m^2	1.000 (reference)	0.846 (0.551 - 1.300)	1.000 (reference)	1.413 (0.935 - 2.135)	1.000 (reference)	1.044 (0.615 - 1.772)	1.000 (reference)	1.021 (0.459 - 2.269)	1.000 (reference)	0.710 (0.443 - 1.136)
≥25 kg/m^2	0.456 (0.235 - 0.883)	0.441 (0.259 - 0.752)	0.530 (0.291 - 0.965)	0.725 (0.435 - 1.208)	0.528 (0.355 - 0.786)	0.624 (0.282 - 1.379)	0.519 (0.354 - 0.760)	0.692 (0.232 - 2.062)	0.452 (0.219 - 0.932)	0.396 (0.228 - 0.687)
*VB_{12}										
≥368 mg	1.000 (reference)	0.991 (0.392 - 2.507)	1.000 (reference)	0.724 (0.312 - 1.679)	1.000 (reference)	0.436 (0.098 - 1.944)	1.000 (reference)	0.606 (0.075 - 4.927)	1.000 (reference)	1.070 (0.402 - 2.846)
< 368 mg	N./A	N./A	N./A	N./A	N./A	N./A	N./A	N./A	N./A	N/A
Triglycerides										
<126.65 mg/dL	1.000 (reference)	0.907 (0.582 - 1.414)	1.000 (reference)	1.532 (1.010 - 2.323)	1.000 (reference)	0.866 (0.512 - 1.465)	1.000 (reference)	0.540 (0.218 - 1.341)	1.000 (reference)	0.662 (0.422 - 1.038)
≥126.65 mg/dL	0.432 (0.241 - 0.775)	0.550 (0.341 - 0.890)	0.668 (0.401 - 1.111)	0.717 (0.465 - 1.106)	0.582 (0.414 - 0.820)	0.436 (0.228 - 0.836)	0.553 (0.401 - 0.765)	0.479 (0.201 - 1.143)	0.255 (0.131 - 0.498)	0.464 (0.290 - 0.742)
Cholesterol										
<150 mg/dL	1.000 (reference)	0.692 (0.261 - 1.837)	1.000 (reference)	1.083 (0.417 - 2.812)	1.000 (reference)	0.618 (0.165 - 2.317)	1.000 (reference)	1.048 (0.243 - 4.524)	1.000 (reference)	0.358 (0.121 - 1.060)
≥150 mg/dL	0.372 (0.147 - 0.940)	0.361 (0.157 - 0.830)	0.344 (0.148 - 0.799)	0.464 (0.217 - 0.992)	0.379 (0.216 - 0.667)	0.548 (0.266 - 1.128)	0.403 (0.234 - 0.694)	0.736 (0.290 - 1.865)	0.261 (0.096 - 0.710)	0.264 (0.106 - 0.659)
HDL										
>42.75 mg/dL	1.000 (reference)	1.070 (0.555 - 2.064)	1.000 (reference)	1.899 (1.010 - 3.569)	1.000 (reference)	1.526 (0.688 - 3.384)	1.000 (reference)	0.383 (0.107 - 1.368)	1.000 (reference)	0.715 (0.358 - 1.424)
≤42.75 mg/dL	2.464 (1.142 - 5.317)	1.865 (0.955 - 3.643)	2.755 (1.379 - 5.507)	2.591 (1.403 - 4.784)	1.930 (1.243 - 2.995)	1.937 (0.883 - 4.248)	1.687 (1.115 - 2.553)	1.268 (0.404 - 3.977)	1.304 (0.554 - 3.070)	1.476 (0.737 - 2.957)
LDL										
<130 mg/dL	1.000 (reference)	0.837 (0.437 - 1.601)	1.000 (reference)	1.358 (0.714 - 2.585)	1.000 (reference)	1.141 (0.496 - 2.625)	1.000 (reference)	N/A	1.000 (reference)	0.707 (0.353 - 1.417)
≥130 mg/dL	0.399 (0.094 - 1.700)	0.207 (0.060 - 0.707)	0.232 (0.052 - 1.042)	0.459 (0.158 - 1.332)	0.230 (0.088 - 0.599)	0.838 (0.200 - 3.518)	N./A	N/A	0.023 (0.001 - 0.402)	0.286 (0.096 - 0.850)

* Folate at 3.8 nmol/L and VB_{12} at 368 mg were the lower 15% cut-offs for each level in colorectal cancer patients and controls.
** Homocysteine at 13.2 μmol/L was the upper 15% cut-off for each level in colorectal cancer patients and controls.
